# Longitudinal Associations between Physical Activity and Affective States: Findings from a 9-Year National Study

**DOI:** 10.1093/geroni/igaf122.2745

**Published:** 2025-12-31

**Authors:** Angela Zhong, Josha Thomas, David Rompilla, Jason Levin, Emily Hittner, Jacquelyn Stephens, Joseph Mikels, Claudia Haase

**Affiliations:** Northwestern University, Evanston, Illinois, United States; Harvard University, Cambridge, Massachusetts, United States; Texas A&M University, College Station, Texas, United States; Northwestern University, Evanston, Illinois, United States; Northwestern University, Evanston, Illinois, United States; Mather Institute, Illinois, United States; DePaul University, Chicago, Illinois, United States; Northwestern University, Evanston, Illinois, United States

## Abstract

Physical activity is widely recognized for its role in boosting positive affect, yet key questions remain regarding its nuanced longitudinal associations with affect. At the micro level, it is uncertain whether benefits extend beyond positive affect to states of varying valence and arousal. At the macro level, it is unclear whether associations generalize across diverse populations. The present study examined longitudinal associations between physical activity and positive and negative affect over nine years using a nationally representative sample of 3,100 Black and White American adults from the Midlife Development in the United States study. Affect was measured using items that varied in valence (positive or negative) and arousal (high or low) from the Positive and Negative Affect Schedule (PANAS) and Negative and Positive Affect Scale (NAPAS). Results indicated that physical activity predicted increases in positive affect over nine years, driven by high-arousal positive affect (e.g., active, enthusiastic), along with reductions in low-arousal negative affect (e.g., hopeless, everything was an effort). These findings controlled for baseline affect, along with health and sociodemographic factors. While most findings generalized across racial groups, effects for positive affect measured by PANAS were specific to White adults. Follow-up analyses probed the generalizability of these findings across different intensity levels of physical activity (light, moderate, vigorous) and across other well-being measures (e.g., life satisfaction). These findings contribute to longstanding discussions about affective benefits of physical activity and underscore the importance of including marginalized populations in research.

